# Sodium valproate and 5-aza-2′-deoxycytidine differentially modulate DNA demethylation in G1 phase-arrested and proliferative HeLa cells

**DOI:** 10.1038/s41598-019-54848-x

**Published:** 2019-12-03

**Authors:** Marina Amorim Rocha, Giovana Maria Breda Veronezi, Marina Barreto Felisbino, Maria Silvia Viccari Gatti, Wirla M. S. C. Tamashiro, Maria Luiza Silveira Mello

**Affiliations:** 1 Department of Structural and Functional Biology, Institute of Biology, University of Campinas (UNICAMP), 13083-862 Campinas, SP Brazil; 2 Department of Genetics, Evolution, Microbiology and Immunology, Institute of Biology, University of Campinas (UNICAMP), 13083-862 Campinas, SP Brazil

**Keywords:** Cell growth, DNA methylation

## Abstract

Sodium valproate/valproic acid (VPA), a histone deacetylase inhibitor, and 5-aza-2-deoxycytidine (5-aza-CdR), a DNA methyltransferase 1 (DNMT1) inhibitor, induce DNA demethylation in several cell types. In HeLa cells, although VPA leads to decreased DNA 5-methylcytosine (5mC) levels, the demethylation pathway involved in this effect is not fully understood. We investigated this process using flow cytometry, ELISA, immunocytochemistry, Western blotting and RT-qPCR in G1 phase-arrested and proliferative HeLa cells compared to the presumably passive demethylation promoted by 5-aza-CdR. The results revealed that VPA acts predominantly on active DNA demethylation because it induced TET2 gene and protein overexpression, decreased 5mC abundance, and increased 5-hydroxy-methylcytosine (5hmC) abundance, in both G1-arrested and proliferative cells. However, because VPA caused decreased *DNMT1* gene expression levels, it may also act on the passive demethylation pathway. 5-aza-CdR attenuated *DNMT1* gene expression levels but increased TET2 and 5hmC abundance in replicating cells, although it did not affect the gene expression of *TETs* at any stage of the cell cycle. Therefore, 5-aza-CdR may also function in the active pathway. Because VPA reduces DNA methylation levels in non-replicating HeLa cells, it could be tested as a candidate for the therapeutic reversal of DNA methylation in cells in which cell division is arrested.

## Introduction

The methylation of cytosine carbon 5, leading to the formation of 5-methylcytosine (5mC), the fifth DNA base, is among the most widely studied epigenetic modifications^1,2^. In mammals, this process is mediated by maintenance DNA methyltransferase 1 (DNMT1) and *de novo* DNMTs 3A and 3B. DNA methylation plays an important role in multiple processes, including genomic imprinting, chromosome X inactivation and heterochromatin formation^3,4^. Aberrant cytosine hypermethylation of certain tumour suppressor gene promoters can be triggered in human cancers, leading to the silencing of these genes and contributing to tumourigenesis^5,6^.

DNA methylation has been long considered to be an epigenetic marker of high stability^7^. A DNA replication-dependent passive process due to DNMT1 inhibition primarily explained changes in its levels. However, events that were not explained by this model, such as the waves of global 5mC loss during the early stages of embryonic development in mammalian cells, suggested that additional demethylating mechanisms may exist^8,9^.

The discovery of 5-hydroxymethylcytosine (5hmC) and ten-eleven-translocation (TET) enzymes in mammalian genomes has opened a new chapter in the field of DNA methylation research^10–12^. The TET family, which comprises the TET1, TET2 and TET3 proteins, has the ability to oxidize 5mC into the cytosine derivatives 5hmC, 5-formylcytosine (5fC) and 5-carboxylcytosine (5caC)^13,14^.

In recent years, biochemical and structural studies have provided mechanistic insights into how TETs and thymine DNA glycosylase (TDG) mediate active DNA demethylation. To complete DNA demethylation, TDG recognizes and excises 5fC and 5caC from the genome, creating abasic sites before unmodified cytosine is restored through base excision repair (BER)^15^. Although several other TET–TDG-independent mechanisms have been proposed to mediate active DNA demethylation, the TET–TDG pathway has been predominantly implicated^16^. The DNA repair machinery can act upon these derivatives, restoring unmodified cytosine and completing the process of active DNA demethylation^17,18^.

There are drugs that directly or indirectly induce DNA demethylation. The cytosine analogues 5-azacytidine (5-aza-CR) and 5-aza-2′-deoxycytidine (5-aza-CdR, decitabine) are classical inducers of passive DNA demethylation that inhibit DNMT1 activity and reduce its abundance^19,20^. Due to their epigenetic effects of reactivating the expression of tumour suppressor genes silenced by DNA methylation, these drugs were approved by the US Food and Drug Administration for the treatment of myelodysplastic syndromes in humans^21^. These cytosine analogues have also demonstrated therapeutic potential in several other types of malignancies, including solid tumours^21^. However, 5-aza-CdR induces greater DNA-hypomethylation compared to 5-aza-CR^21^.

Valproic acid/sodium valproate (VPA), a short-chain fatty acid, is a well-known anticonvulsive drug to treat seizures^22,23^ and is a classical histone deacetylase inhibitor (HDACi)^24,25^. VPA also affects DNA methylation in several cell types, including neuroblastoma^26^, human embryonic kidney HEK 293 cells^27,28^, rat neural stem cells^29^, human hepatocytes^30^, human hepatocellular carcinoma HepG2 cells^31^ and human cervical carcinoma HeLa cells^32^. The epigenetic changes introduced by VPA affect expression of genes related to cell differentiation, growth inhibition and apoptosis^33^. In phase I and II clinical trials, this drug exhibited antitumour potential^34–37^. VPA is also a successful therapeutic compound when combined with other chemotherapy agents^37–40^.

The novelty regarding the functional activities of both DNMT and HDAC inhibitors was the observation that, in addition to their consolidated mechanisms of action, these agents might also act on active DNA demethylation pathways. While changes in the levels of cytosine derivatives have already been described in response to 5-aza-CR and 5-aza-CdR, studies of VPA and another HDACi, Trichostatin A, were focused on the drug-induced DNA demethylation process independent of DNA replication^27,41–45^.

In HeLa cells, DNA demethylation was observed in response to VPA treatment and was shown to contribute to the chromatin remodelling previously assumed to be caused by HDAC inhibition^32,46^. Although DNA methylation alterations are reversible, they are more stable than histone acetylation changes, and long-term consequences to cellular programmes could thus be induced by exposure to VPA^28^.

To understand whether VPA-induced DNA demethylation is dependent on a passive DNA replication pathway, the investigation of both dividing and non-proliferative cells is crucial. For this reason, cell synchronization is necessary because it allows for investigations involving cell proliferation and can produce enriched populations at different stages of the cell cycle^47^. Several methods have been developed to arrest cells in the G1 phase, including the inhibition of DNA synthesis by lovastatin. This treatment induces non-toxic cell synchronization and is followed by mevalonate treatment, which is necessary to stimulate cell division^48^. Mevalonate induces synchronous recovery of DNA synthesis after a certain latency period (15–18 h), allowing cells to be obtained for analysis at different cell cycle time points, including during the S phase^49^.

In this study, the extent of epigenetic changes on active DNA demethylation elements induced by VPA compared to those induced by 5-aza-CdR treatment were investigated in non-proliferative (G1 phase) and proliferative (S phase) HeLa cells. Our objective was to elucidate how VPA and 5-aza-CdR might elicit alterations in the dynamic and intricate DNA methylation regulatory pathways in these cells.

## Results

### VPA and 5-aza-CdR affect DNA methylation in different phases of the HeLa cell cycle

**Figure 1:**
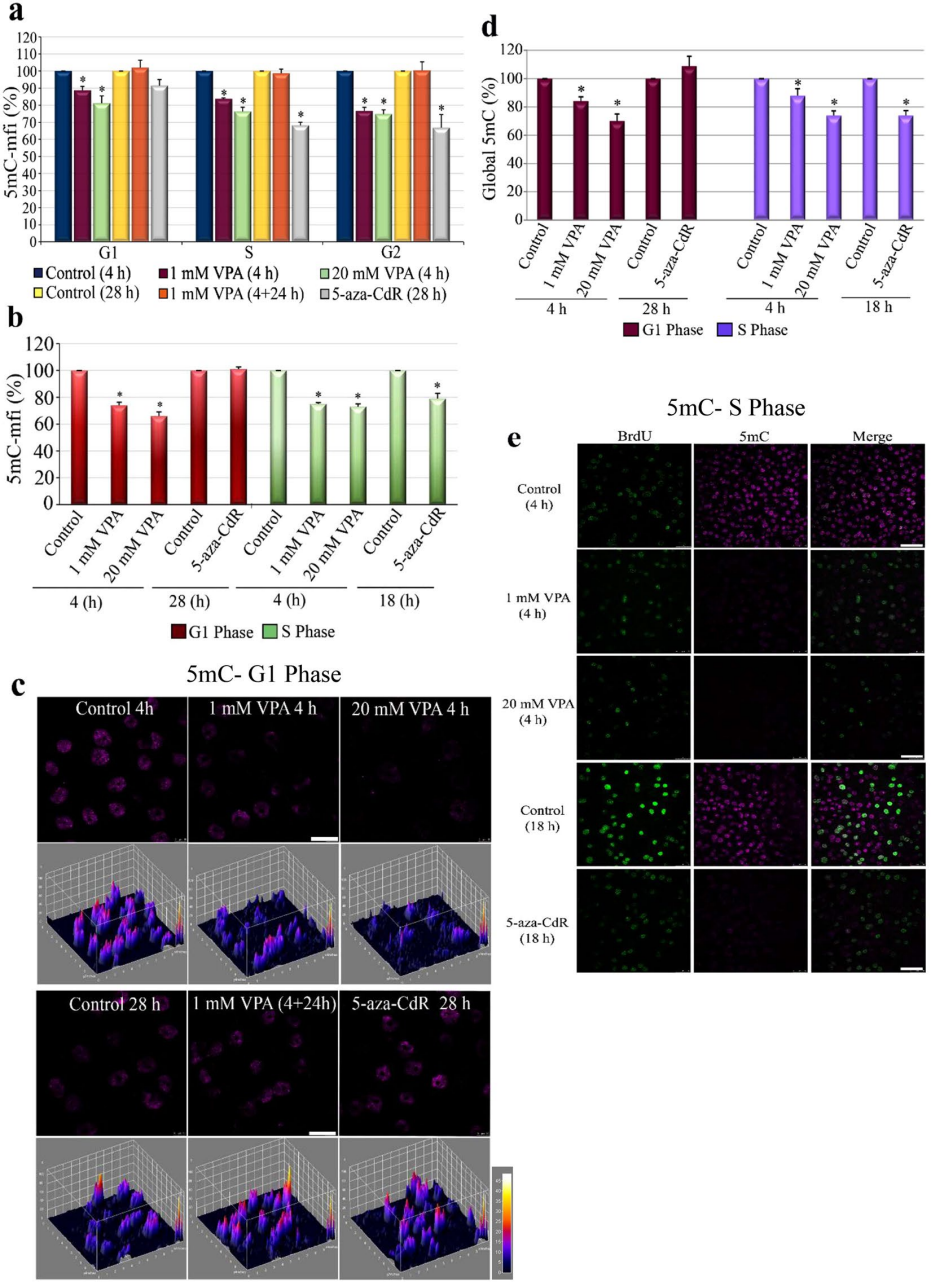
5mC global levels in synchronized and non-synchronized HeLa cells analysed by flow cytometry (**a,b**), ELISA (**d**) and confocal microscopy (**c,e**). Non-synchronized cells were analysed for 5mC content according to their cell cycle phases. 5mC/DNA data were calculated from the mean fluorescence intensity (mfi) of 5mC vs. propidium iodide (PI) (**a**). The 5mC content was also analysed in synchronized cells collected in the G1 (**b–d**) and S (**b,d,e**) phases. Using Student’s *t*-test, significant differences at P < 0.05 between the treatments and respective controls are indicated (*). A significant decrease in 5mC in VPA- treated, G1-arrested cells under non-proliferative (**b–d**), proliferative (**b,d,e**) and non-synchronized (**a**) cells was observed for all assays. After treatment with 5-aza-CdR, this decrease was observed only in cells collected or selected from the S phase (**a,b,d,e**). Data are representative of three independent experiments for flow cytometry and ELISA and two independent experiments for confocal microscopy. Scale bars indicate 20 μm.

The exposure of non-synchronized HeLa cells to 1 and 20 mM VPA for 4 h promoted a reduction in the levels of 5mC in the G1, S and G2 phases [Fig. 1a]. The same result was observed in synchronized cells that were arrested in the G1 phase [Fig. 1b–d] and in proliferative cells collected in the S phase [Fig. 1b,d,e]. However, in cells treated with 5-aza-CdR, the reduction in 5mC was only observed in replicating cells. These results were demonstrated using flow cytometry, ELISAs, and confocal microscopy. To investigate the duration of these VPA-induced alterations, non-synchronized cells treated with 1 mM VPA were subsequently cultivated in the absence of the drug and analysed for the same parameters as cells treated for 4 h. The levels of 5mC were completely reversed within the first 24 h of this treatment [Fig. 1a].

### Both VPA and 5-aza-CdR treatments induce increased abundance of the active DNA demethylation intermediate 5 hmC

**Figure 2:**
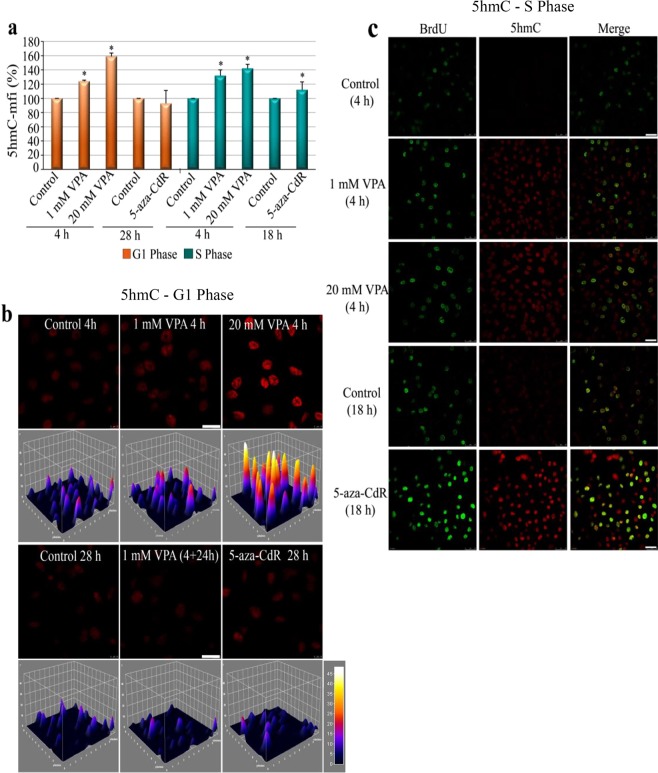
5hmC after VPA and 5-aza-CdR treatments using flow cytometry and confocal microscopy. The abundance of 5hmC increased in HeLa cells at the G1 (**a,b**) and S (**a,c**) phases after VPA treatment, but only in proliferating cells after 5-aza-CdR treatment (**a,c**). 3D graphs plotted from representative images revealed fluorescence intensity based on an 8-bit intensity scale (0–110) (**b**). Images are representative of two experiments for confocal microscopy and three experiments for flow cytometry. Using Student’s *t*-test, significant differences at P < 0.05 between treatments and the respective controls are indicated (*). Scale bars indicate 20 μm.

Considering that a short duration of VPA treatment induced 5mC loss, we next investigated whether this drug affected global 5hmC levels, which are related to active DNA demethylation pathways. Using flow cytometry and confocal microscopy, the fluorescence intensity of signals for 5hmC was observed to significantly increase after VPA treatment in both G1-arrested [Fig. 2a,b] and proliferating cells [Fig. 2a,c]. However, in cells treated with 5-aza-CdR, increased 5hmC levels were only detected in proliferating cells [Fig. 2a,c].

### VPA and 5-aza-CdR treatments do not elicit visually observable changes in 5fC and 5caC abundance or distribution in synchronized HeLa cells

Because 5hmC abundance was altered after the VPA and 5-aza-CdR treatments, the 5fC and 5caC markers were also expected to be affected. However, when these 5mC derivatives were investigated using immunofluorescence, their signals were found to be homogeneously distributed throughout the nuclei in both the treated and control cells. Differences in the fluorescence intensity between the treated and control cells arrested in the G1 phase were not observed [Supplementary Fig. S1a,b].

### VPA and 5-aza-CdR affect gene expression related to key proteins involved in cytosine methylation and demethylation pathways

**Figure 3:**
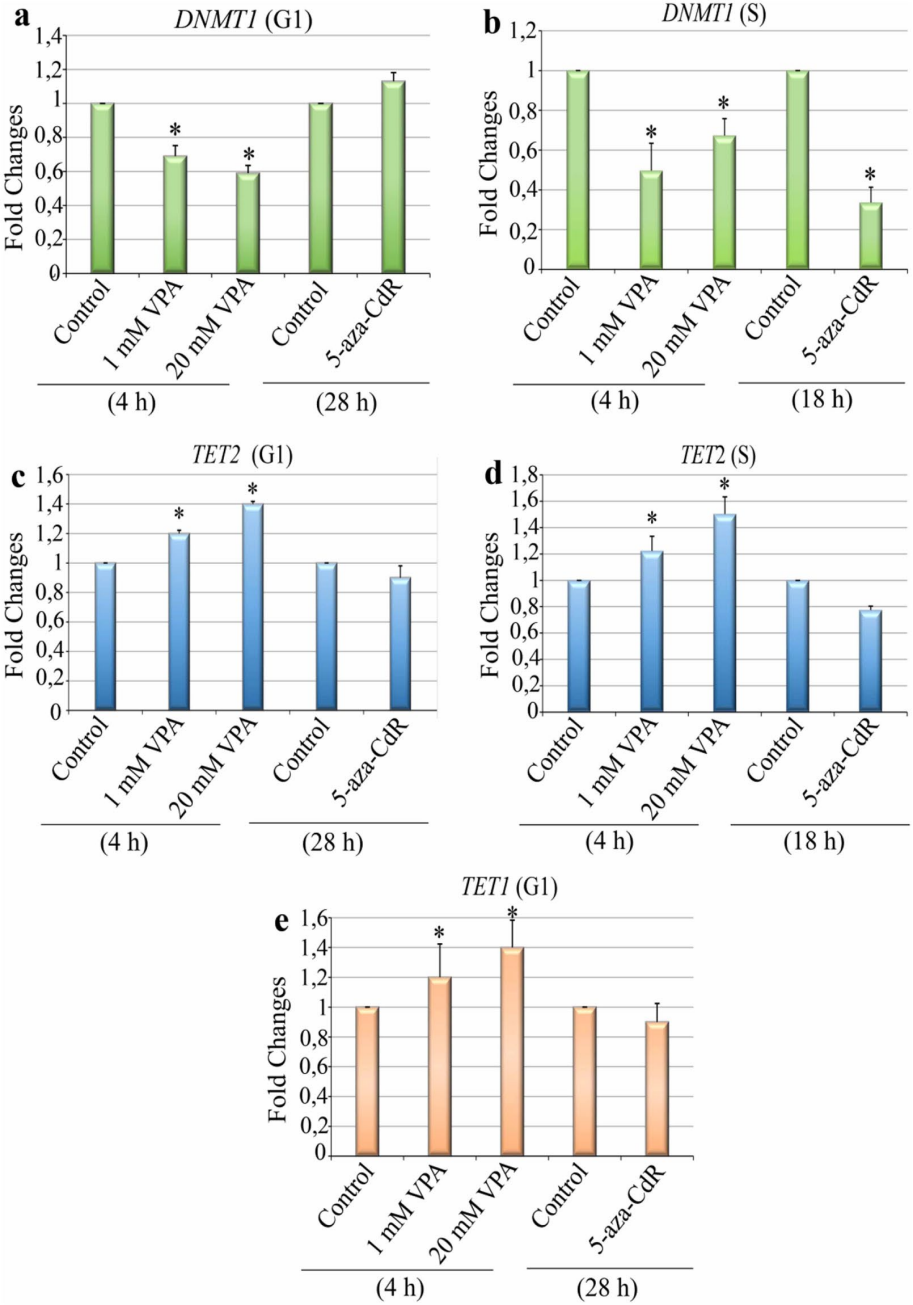
Gene expression of *DNMT1*, *TET1* and *TET2* in VPA- and 5-aza-CdR- treated, synchronized HeLa cells. VPA induced decreased *DNMT1* gene expression levels in cells at the G1 and S phases, whereas 5-aza-CdR only promoted this decrease in cells in the S phase (**a,b**). The *TET2* gene expression was altered after treatments with 1 and 20 mM VPA in both phases of the cell cycle but remained unaltered in response to 5-aza-CdR (**c,d**). *TET1* gene expression levels increased after VPA treatment in the G1-arrested cells (**e**). Data obtained from RT-PCR analyses were normalized to endogenous *GAPDH* controls. Using Student’s *t*-test, significant differences at P < 0.05 between the treatments and respective controls are indicated (*). Data are representative of three independent experiments.

The present results suggest a role for the active DNA demethylation process in HeLa cells in response to both VPA and the classical passive demethylating agent 5-aza-CdR. Consistent with this hypothesis, we observed that both of these drugs modulated the gene expression of the DNMT1, TET1 and TET2 enzymes, which are directly involved in the dynamics of cytosine derivatives. The mRNA expression levels of *DNMT1*, which is involved in DNA methylation maintenance, were significantly reduced in response to treatment with 1 and 20 mM VPA in proliferating and G1-arrested cells. However, after 5-aza-CdR treatment, this reduction was only verified in proliferating cells [Fig. 3a,b]. Unexpectedly, for cells in S phase, a more substantial reduction in *DNMT1* expression was observed after treatment with the lower VPA concentration. Increased *TET2* gene expression was observed after VPA treatment in both the G1 and S phases of the cell cycle. In contrast, mRNA expression levels of this enzyme induced by 5-aza-CdR remained unchanged [Fig. 3c,d]. In G1-arrested cells, expression level of the *TET1* gene increased only after VPA treatment [Fig. 3e].

### VPA and 5-aza-CdR differentially affect the protein levels of DNMT1, TET2 and TDG

**Figure 4:**
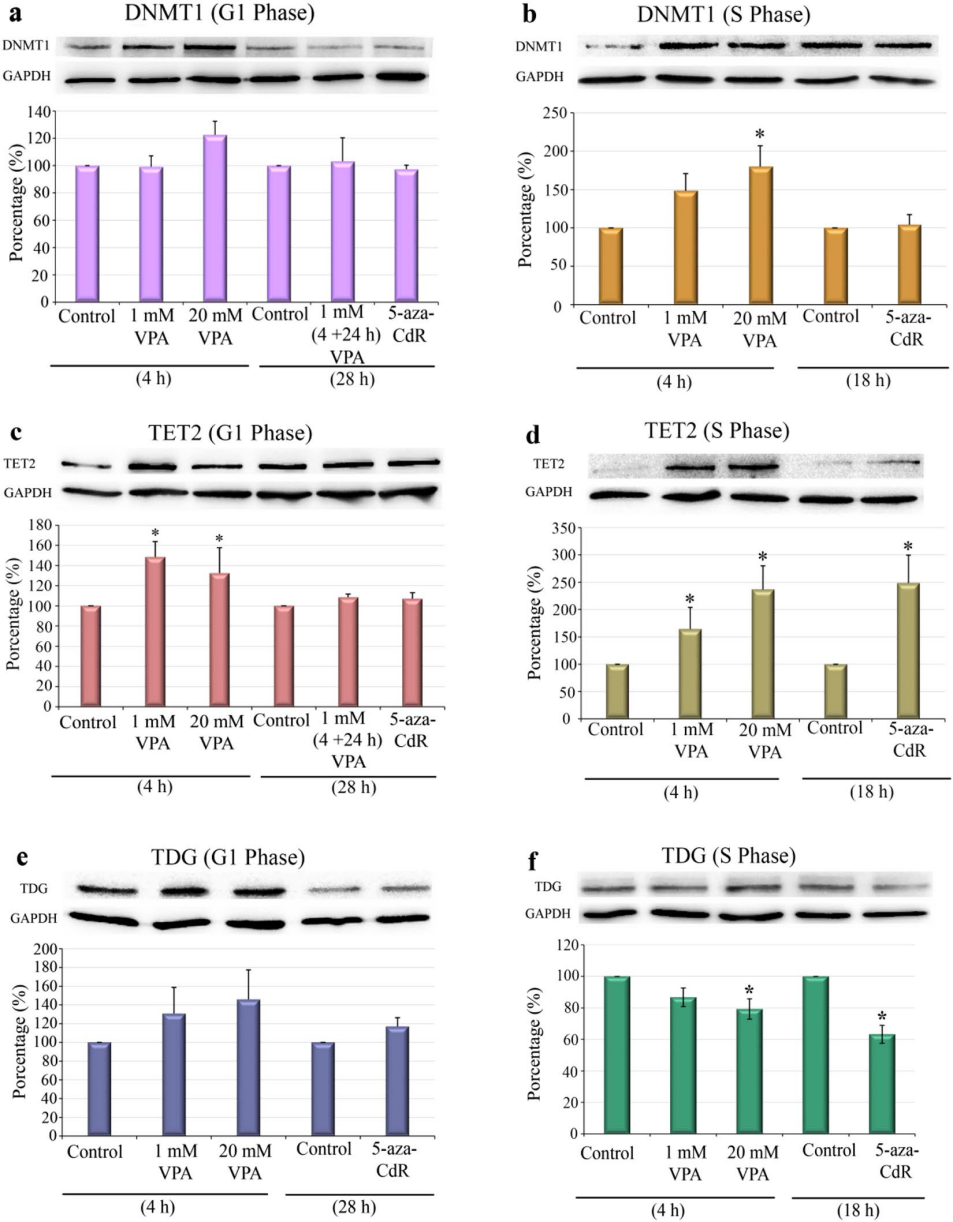
DNMT1, TET2 and TDG protein levels in VPA- and 5-aza-CdR-treated, synchronized HeLa cells. Western blots and densitometry quantifications of DNMT1, TET2 and TDG are shown. The DNMT1 levels increased compared to the respective controls after treatment with 20 mM VPA in proliferating cells (**b**) but not in G1-arrested cells (**a**). The TET2 protein levels were significantly affected by treatment with 1 mM and 20 mM VPA compared to the respective controls in G1-arrested (**c**) and proliferating cells (**d**) and by treatment with 5-aza-CdR in proliferating cells (**d**). The TDG protein levels decreased in replicating cells (**f**) but not in G1-arrested cells (**e**) after cell exposure to 20 mM VPA and 5-aza-CdR. GAPDH was used as a loading control. Using Student’s *t*-test, significant differences at P < 0.05 between treatments and respective controls are indicated (*). Three to seven independent experiments were performed, and blots were processed in parallel. The grouping of blots including the loading control and specific proteins, was derived from the same experiment. Full-length blots are included in the Supplementary Information file [Supplementary Fig. S5].

**Figure 5:**
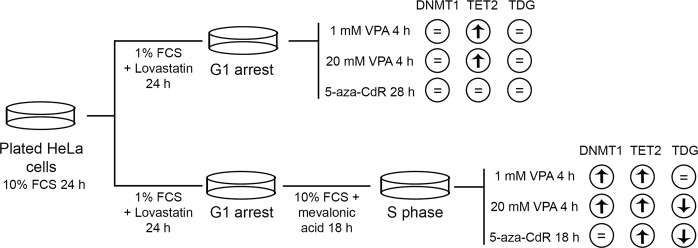
Schematic representation summarizing the main results shown in Fig. 4.

Western blotting and densitometry analysis showed that, in contrast to the gene expression data, the DNMT1 abundance increased in proliferating cells that were in S phase, but not in cells that were arrested in G1 phase, when treated with 20 mM VPA [Fig. 4a,b]. Regarding the effect of VPA on TET2 levels, changes were observed irrespective of drug concentration in both G1-arrested and proliferating cells. In these cases, TET2 levels increased compared to the respective controls [Fig. 4c,d].Unexpectedly, the increased TET2 content was also observed in proliferating cells treated with 5-aza-CdR [Fig. 4d]. Because TDG has been proposed to participate with TET in active DNA demethylation, TDG was also investigated. In the G1-arrested cells, no change in TDG abundance was induced by VPA or 5-aza-CdR [Fig. 4e], whereas in the replicating cells, there was a significant reduction in this protein, when HeLa cells were exposed to 20 mM VPA and 5 μM 5-aza-CdR [Fig. 4f]. These findings are schematically summarized in Fig. 5.

## Discussion

VPA has several mechanisms of action, including acting as an HDAC inhibitor^24,25^, a DNA global demethylator^26,27,29–32^, histone methylator and/or demethylator^50,51^, and contributor to the process of chromatin remodelling^46^. The mechanism for the removal of 5mC from DNA remains poorly understood^52^. Therefore, this study aimed to investigate which pathway, active or passive, causes the loss of 5mC from DNA, when induced by VPA in HeLa cells. For this purpose, DNA demethylation in HeLa cells was investigated under similar conditions between cells whose cell cycles were arrested in G1 phase and proliferating cells collected in S phase.

The results revealed that VPA plays a key role in the active DNA demethylation pathway in HeLa cells. This phenomenon was previously proposed, although it was reported for non-synchronized cells^32^. Using cellular synchronization and VPA treatments, the overexpression of *TET1* and *TET2* genes, the decreased abundance of 5mC and the increased abundance of 5hmC were demonstrated, even with the arrest of cell proliferation. Thus, the active demethylation pathway induced by VPA in HeLa cells was elucidated in detail. However, VPA also altered the expression profile of DNMT1, which is a novel observation, demonstrating that VPA may affect both active and passive demethylation processes.

The results suggesting that VPA induces DNA demethylation in non-replicating cells in a dose-dependent manner have been described in previous studies involving the transient transfection of methylated plasmids into the cell, demonstrating that demethylation occurs independently of DNA replication^27^. In addition, VPA was shown to potentially reverse DNA methylation patterns in non-dividing cells^27^. Therefore, our data may have important potential implications for the therapeutic reversal of DNA methylation in tissues in which there is no further involvement of cell division.

Modifications in DNA methylation may result in long-term consequences. The durability of VPA-induced DNA demethylation was investigated in synchronized G1-arrested cells, demonstrating that abundance of the 5mC markers was completely reversed after the first 24 h of cell culture in the absence of drug treatment. This result differs from those of a previous report in non-synchronized cells^32^ but confirms that changes in DNA methylation are reversible^28^. To better understand how long VPA maintains these modifications in DNA methylation status in synchronized cells, it will be necessary to vary the duration of time that cells are cultured in the absence of the drug following drug treatment.

From our results for 5fC and 5caC methylated cytosine derivatives, the observation that global levels of these molecules did not change after either VPA or 5-aza-CdR treatment under the experimental conditions in G1-arrested cells does not necessarily indicate that these derivatives are not affected. Both 5fC and 5caC cytosine derivatives are naturally scarce markers, primarily because they are direct targets of the TDG/BER mechanism that rapidly replaces them with unmethylated cytosine^15,17,52^. When the association between TDG protein and the 5fC and 5caC abundance was analysed in HeLa cells in response to treatment with VPA or 5-aza-CdR, no significant association was observed in G1-arrested cells. However, a significant decrease in the TDG protein levels was observed in 20 mM VPA- and 5-aza-CdR-treated proliferating cells harvested during the S phase. Previous reports have proposed that the steady state of 5fC and 5caC may not accurately reflect whether demethylation is occurring due to their low abundance or because they are recognized and rapidly removed by mechanisms involving the TDG protein^52^. To address this issue in mouse embryonic stem cells and mouse embryonic fibroblasts, the conditional deletion of *TDG* has demonstrated an accumulation of 5fC/5caC^53^. Although our results did not show a significant increase in TDG abundance in VPA-treated proliferating cells compared to controls, Western blotting results showed the presence of this protein, suggesting a possible association between 5fC/5caC and TDG.

Although the *DNMT1* gene expression decreased following VPA treatment in both G1 and S cell cycle phases, the DNMT1 abundance increased in 20 mM VPA-treated proliferating cells. These results seem to be contradictory, especially considering that previous reports have shown increased *DNMT1* gene expression but decreased DNMT1 abundance (human lens epithelial cells) or a depletion in DNMT1 gene and protein expression levels (breast cancer cells) following VPA-dependent DNA demethylation^54,55^. However, these studies involved longer VPA treatments (2- to 3-day duration). Perhaps the 4-h VPA treatment duration used in the present study was not sufficient to cause these types of alterations. Alternatively, the results in this study may reflect a particular response feature of HeLa cells to VPA. Our results further showed that 5-aza-CdR removes 5mC markers from DNA by interfering with DNMT1 maintenance in cells undergoing DNA replication. Of note, DNMT1 affects the activity of three important suppressor genes in HeLa cells^56^, and increased expression levels of this protein are associated with progressive severity of the cervical lesions^57^.

DNMT1 can provide binding sites for methyl-binding domain proteins, which can mediate gene expression through interactions with HDACs. Regarding the VPA action on DNMT1, it may be hypothesized that because VPA is a classic HDAC inhibitor, it may promote structural and functional changes in DNMT1 if affecting the HDAC2 that links to the N-terminal domain of DNMT1, in addition to linking to the DNMT1 C-domain^58,59^. This effect would interfere with the DNMT1 function of inducing cytosine methylation at the S phase^58^.

Lower *DNMT1* gene expression following VPA treatment may indicate that this drug prevented transcription factors (TFs) from accessing the required target-binding sites for such a gene to be transcriptionally activated. In NIH3T3 cells, *DNMT1* transcription is regulated by TFs Sp1 and Sp3. Sp1 is expressed predominantly at the G1 phase and Sp3 is expressed at the S phase^60^. Sp3 activation requires involvement of the p300 co-activator^60^. Other TFs, including E2F, have also been reported to be involved in *DNMT1* expression^61^. Whether any of these proteins are affected by VPA leading to the decreased *DNMT1* gene expression detected in HeLa cells is unknown and requires investigation.

Because histone methylation may work independently or in concert with DNA methylation^62^, whether VPA also affects the repressive trimethylation of lysine 27 at histone H3 (H3K27me3) is also a matter of interest for further studies. The PcG protein EZH2 (enhancer of Zeste homologue 2) interacts with DNMTs in HeLa cells^63^. EZH2 catalyses the addition of -CH_3_ groups to lysine 27 at histone H3, contributing to the formation of a repressive chromatin state^64^. Whether VPA affects the participation of EZH2-mediated H3K27me3 in this process remains to be clarified.

Our findings indicated that in addition to VPA reducing overall levels of 5mC, it also promotes increased levels of 5hmC, regardless of whether cells are replicating their DNA content. To date, the only biological mechanism shown to generate 5hmC and 5fC by 5mC oxidation involved members of the TET family of enzymes^16^ which in turn, have been affected in response to VPA.

The upregulation of TET2 expression and the increased abundance of cytosine derivatives have also been found in hepatocellular cancer cells and human skin fibroblasts in response to 5-aza-CR^44,45^. Interestingly, although 5-aza-CR shares many structural and functional similarities with 5-aza-CdR, these compounds have opposite effects on TET2, where HeLa cells have unchanged gene expression but increased protein expression levels during the S phase. The latter may be justified by the possibility that the DNA demethylation process promoted by aza-CdR depends on cell proliferation.

Although the negative regulation of *TET2* gene expression concomitant with the increased abundance of 5hmC promoted by 5-aza-CdR in HeLa cells may seem controversial, a recent study of myeloid leukaemia treated with 5-aza-CdR described globally reduced levels of 5mC and increased levels of 5hmC, 5fC and 5caC, without altering TETs^43^. One explanation for this observation is that if TET enzymes preferentially act on hemi-methylated CpG, 5-aza-CdR may alter the 5hmC, 5fC and 5caC levels through the increased recruitment of TETs without causing changes in their levels, which would support our observed results for HeLa cells.

The lack of association between TETs and 5hmC abundance has also been reported in rectal cancer cells, where decreased TET1 but increased 5hmC was observed, suggesting that these effects may occur through a mechanism not yet elucidated since the mechanism of action of the TETs remains poorly understood^17,65^. In addition, the observation of increased DNA-bound TETs after 5-aza-CdR treatment suggests a change in TET dynamics and their consequent enzymatic activity after drug administration, suggesting that they are one of the factors responsible for the increased abundance of 5mC and their derivatives^43^.

In HeLa cells, VPA may initially induce passive demethylation by the suppression of DNMT1 activity. In addition, VPA may induce active demethylation, independent of replication, causing the expression of *TET1* and *TET2* and the abundance of one of the active DNA demethylation pathway intermediates (5hmC) to be noticeably increased, leading to decreased overall DNA methylation. A previous report has shown that VPA can act on these two DNA demethylation pathways in human lens epithelial cells^54^. In this case, DNMT1, DNMT3a, DNMT3b and TET1 were shown to be altered in the expression of the passive DNA demethylation pathway enzymes^54^.

## Conclusion

The results from this study indicate the complexity of the DNA demethylation process in HeLa cells, either in response to VPA or 5-aza-CdR, suggesting that these compounds do not act through a single pathway. However, these effects that contribute to the removal of 5mC, which is not apparent in 5-aza-CdR-treated G1-arrested HeLa cells, are indicative of the dependence of this drug activity on the DNA replication process to act through the passive pathway. Nevertheless, the components of the active pathway could also be affected during this process, which is consistent with reports for some cell lines. In contrast to 5-aza-CdR activity, VPA promotes DNA demethylation effects on HeLa cells independent of cell proliferation in G1-arrested cells, confirming the participation of VPA in the active demethylation process, although passive pathway components may also be affected. Although the present investigation was conducted using only one cell type, the fact that VPA reduces DNA methylation levels in non-replicating HeLa cells suggests the hypothesis that VPA could be a candidate for the therapeutic reversal of DNA methylation in cells where cell division is arrested.

## Materials and Methods

### Cell culture and VPA and 5-aza-CdR treatments

HeLa cells acquired at passage 126 from the Instituto Adolfo Lutz (São Paulo, Brazil), which were originally purchased from ATCC CCL-2 (ATCC, Manassas, VA, USA) were used at passages 210–230. The cells were incubated at 37 °C in a 5% CO_2_ atmosphere and grown in Dulbecco’s modified Eagle’s medium (DMEM) (Sigma-Aldrich^®^, St. Louis, MO, USA) with 10% bovine calf serum (FCS) (Cultilab^®^, Campinas, SP, Brazil), penicillin/streptomycin (Sigma-Aldrich^®^) (100 IU and 100 μg/mL, respectively) and 1% sodium pyruvate (Sigma-Aldrich^®^). For flow cytometry, and for DNA, RNA and protein extraction, the cells were cultivated at 1.5 × 10^5^ cells/mL in 6-well plates. For immunofluorescence studies, the cells were seeded into 24-well plates onto round coverslips at 5 × 10^4^ cells/mL. After a 24-h of cultivation in complete medium (10% FCS), non-synchronized and synchronized cells collected in the G1 phase were cultivated in 1% FCS medium containing sodium valproate (VPA) (Sigma-Aldrich^®^) at a concentration of 1 or 20 mM for 4 h^32^ or 5-aza-CdR (Millipore^®^, Billerica, MA, USA) at 5 μM for 28 h. Cells treated with 1 mM VPA for 4 h and then cultured for 24 h in the medium without the drug were also used. For synchronized cells collected in the S phase, treatments were performed with 1 and 20 mM VPA for 4 h and with 5 μM 5-aza-CdR for 18 h. The concentration and time of treatment with 5-aza-CdR were adapted from a previous report^43^. For the controls, cells were cultivated in the absence of the drugs. An MTT assay showed that VPA and 5-aza-CdR did not reduce cell viability under the above-mentioned treatment conditions [Supplementary Fig. S2a].

### Cell synchronization

For optimal cell concentration to obtain cells arrested in the G1 phase, HeLa cells were initially plated and then cultured in fresh 1% FCS medium containing 20 μM lovastatin (Sigma-Aldrich^®^) for 24 h. In another assay, the cells were arrested in the G1 phase and then washed with PBS and induced to continue cycling by replacing the medium with 10% FCS containing 6 mM mevalonic acid (Santa Cruz Biotechnology^®^, Dallas, TX, USA) for 18 h to obtain cells in the S phase. The results obtained with the MTT assay showed that lovastatin did not reduce the cell viability under the treatment conditions used [Supplementary Fig. S2b]. Cell synchronization was preceded by preliminary assays based on adaptations from published protocols^48,49^ [Supplementary Fig. S3].

### Combined 5mC and 5hmC quantification and cell cycle analysis using flow cytometry

The cell cycle profiles of non-synchronized and synchronized cells immunolabelled with 5mC and 5hmC were analysed by flow cytometry by modifying a previously reported protocol^28^. Briefly, the cells were washed with PBS supplemented with 0.1% Tween 20 and 1% bovine serum albumin (PBST-BSA) and fixed with 0.25% paraformaldehyde at 37 °C for 10 min and 88% methanol at -20 °C for 30 min. After two washes in PBST-BSA, the cells were treated with 2 N HCl at 37 °C for 30 min, 0.1 M sodium borate buffer solution (pH 8.5) for 5 min and then blocked with 10% BSA (Sigma-Aldrich^®^) in PBST-BSA for 20 min at 37 °C. Next, the cells were incubated with mouse anti-5-mC (1: 200 dilution) (Sigma-Aldrich^®^) and rabbit anti-5hmC (1: 2000 dilution) (Active Motif^®^, Carlsbad, USA) primary antibodies in blocking solution overnight at 4 °C protected from light, followed by extensive PBS washes. To detect primary antibodies, goat anti-mouse conjugated to FITC (1:50 dilution) (Sigma-Aldrich^®^) and goat anti-rabbit conjugated to Alexa-Fluor 488 (1:1000 dilution) (Life Technologies^®^, Carlsbad, CA, USA) were used as secondary antibodies for 1 h in the dark. Staining with 40 μg/mL propidium iodide (PI) (Sigma-Aldrich^®^) in the presence of 10 μg/mL RNase (Thermo Scientific^TM^, Carlsbad, CA, USA) was performed 30 min after secondary antibody rinsing to allow DNA analysis during the cell cycle. Finally, the cells were washed three times and suspended in PBS. The cell cycle profile was determined using a FACS Canto II flow cytometer (BD Biosciences, San Jose, CA, USA) equipped with the BD Diva™ FACS software at the Central Laboratory of High Performance Life Sciences Technologies (LaCTAD, Unicamp, Campinas, SP, Brazil). Analyses were performed as previously reported^66^. Briefly, to exclude debris and aggregates or dead cells, the cells were evaluated in the *Side Scatter* (SSC)/*Forward Scatter* (FSC) dot-plot using the FlowJo® software. Subsequently, fluorescence was quantified based on the mean fluorescence intensity (mfi). A ratio was calculated between the intensity of 5mC or 5hmC fluorescence and PI-DNA fluorescence, using the FlowJo® software [Supplementary Fig. S4].

### Immunofluorescence assays

Synchronized cells adhered to round glass coverslips were collected in the G1 phase and were fixed in absolute methanol at −20 °C for 10 min, rinsed in PBS, and subjected to treatment in 2 N HCl solution for1 h at 37 °C. After two washes in borate buffer (100 mM boric acid, 75 mM NaCl and 25 mM sodium tetraborate, pH 8.5), the preparations were blocked with 1% BSA in PBS for 30 min. Next, the cells were incubated overnight with mouse anti-5mC (1: 100 dilution) (Sigma-Aldrich®), rabbit anti-5hmC (1: 2000 dilution) (Active Motif®), rabbit anti-5caC (1: 500 dilution) (Diagenode®, Liege, Belgium) and rabbit anti-5fC (1: 500 dilution) (Active Motif®) primary antibodies in blocking solution at 4 °C and protected from light, followed by extensive PBS washes. To detect primary antibodies, an anti-mouse antibody conjugated to Alexa-Fluor 647, (1: 250 dilution) (Life Technologies®) and an anti-rabbit antibody conjugated to Alexa-Fluor 647 (1: 1000 dilution) were used as secondary antibodies for 1 h in the dark. For cells whose cell cycle was arrested in the S phase, 10 mM bromo-deoxyuridine (BrdU) (Sigma-Aldrich®) was added to the culture medium, and the cells were incubated for 1 h at 37 °C. Then, the cells were immunolabelled as reported above. The primary antibodies used were anti-5mC and anti-5hmC, and the secondary antibody was used as reported above. Finally, incubation with anti-BrdU-FITC (1:50 dilution) (Abcam, Cambridge, UK), was performed for 1 h in the dark. The samples were analysed using a Leica TCS SP5 II confocal microscope (Wetzlar, Germany) at the LaCTAD facilities. Imaging was performed under the same exposure conditions for all treatments and was analysed using the interactive 3D surface plot plugin in ImageJ software (NIH, Bethesda, MD, USA).

### ELISA for 5mC quantification

The genomic DNA from cells arrested in the G1 and S phases was isolated using a QIAamp DNA mini kit (Qiagen®, Hilden, Germany) and quantified using Nanodrop 2000 equipment (Thermo Scientific^TM^). Concomitant to the concentration analysis, a 260/280 nm ratio of approximately 1.8 was detected for the samples, indicating extraction purity according to the manufacturer’s instructions. Equal concentrations of extracted DNA (100 ng) per sample were used in the 5-methylcytosine DNA ELISA Kit (Zymo®, Irvine, CA, USA). Quantification of 5mC levels was performed according to the manufacturer’s instructions.

### Quantitative real-time RT-PCR

For gene expression analysis, VPA- and 5-aza-CdR-treated cells collected in the G1 and S phases were subjected to total RNA isolation using an RNeasy mini kit (Qiagen®) followed by cDNA production using a High-Capacity cDNA Reverse Transcription kit (Applied Biosystems®, Hilden, Germany). Nanodrop equipment was used to assess the quality of the isolated RNA. RT-PCR reaction plates were prepared using TaqMan Universal PCR Master Mix (Applied Biosystems®), the produced cDNA and TaqMan Gene Expression Assays (Applied Biosystems®) for *DNMT1* (Hs00945875_m1), *TET1* (Hs00286756_m1) and *TET2* (Hs00325999_m1), with *GAPDH* (Hs02758991_g1) as the endogenous control. PCR was conducted on an Applied Biosystems 7500 real-time PCR system (Applied Biosystems®), and cycle threshold (Ct) values were calculated based on experiments performed in triplicate and normalized with respect to the endogenous GAPDH control gene.

### Western blotting

Total protein extraction of cells collected in the G1 and S phases was performed using RIPA buffer (50 mM Tris-HCl (pH 8.0), 150 mM NaCl, 1% Triton X-100; 0.5% sodium deoxycholate, 0.1% SDS, 1 mM EDTA, 0.5 mM EGTA and 1 mM PMSF) for at least 30 min on ice. A Bradford assay (Sigma-Aldrich^®^) was used to determine protein concentrations using bovine serum albumin as a standard. Absorbance values of all samples were measured after incubation for 1 h at room temperature at λ = 595 nm in a Multiskan™ FC Microplate Photometer (Thermo Scientific™). Forty micrograms of protein were loaded into SDS-PAGE gels at different percentages, each for the separation of a specific protein of interest (12% for TDG, 8% for DNMT1 and a 4–10% gradient for TET2), ran on a Tris-glycine buffer system, and transferred to a nitrocellulose membrane (Thermo Scientific^®^). The membranes were blocked for 2 h in 4% milk buffer and incubated, separately, with the corresponding rabbit anti-TDG (1:2000 dilution) (Thermo Scientific^®^), rabbit anti-DNMT1 (1:2000 dilution) (Cell Signaling Technology^®^, Danvers, USA) and rabbit anti-TET2 (1:1000 dilution) (Cell Signaling Technology^®^) primary antibodies overnight in blocking solution at 4 °C. After extensive washes, the membranes were incubated with an anti-rabbit secondary antibody conjugated to peroxidase (1:5000 dilution) (Chemicon^®^, Billerica, MA, USA) in blocking solution for 2 h, followed by several washes. Protein blots were imaged using an ECL Western blotting detection system (Amersham^®^, Pittsburgh, PA, USA) and visualized by chemiluminescence with an Alliance 6.7 imaging system (UVITEC, Cambridge, UK) at the Obesity and Comorbidities Research Centre (University of Campinas, Brazil). Glyceraldehyde 3-phosphate dehydrogenase (GAPDH) was used as the loading control. TDG/GAPDH, DNMT1/GAPDH, and TET2/GAPDH ratios were obtained using ImageJ software. The results shown are representative of four independent experiments.

### Statistical analysis

Data are presented as the mean ± standard error (SE). Differences between controls and treatments in assays, such as quantification by flow cytometry and gene and protein expression were analysed using the Student’s *t*-test. Values of P < 0.05 were considered statistically significant.

## Supplementary information

Supplementary Figures
