# Supplementary material for: Sodium valproate and 5-aza-2′-deoxycytidine differentially modulate DNA demethylation in G1 phase-arrested and proliferative HeLa cells
Source: Sci Rep. 2019 Dec 3;9:18236. doi: 10.1038/s41598-019-54848-x (PMC6890691; doi:10.1038/s41598-019-54848-x)
Supplement: Supplementary file 1 — Supplementary Figures [file 41598_2019_54848_MOESM1_ESM.docx]

**Supplementary Information**

**Sodium valproate and 5-aza-2’-deoxycytidine differentially modulate DNA demethylation in G1 phase-arrested and proliferating HeLa cells**

Marina Amorim Rocha^1^, Giovana Maria Breda Veronezi^1^, Marina Barreto Felisbino^1^, Maria Silvia Viccari Gatti^2^, Wirla M. S. C. Tamashiro^2^, Maria Luiza Silveira Mello^1*^


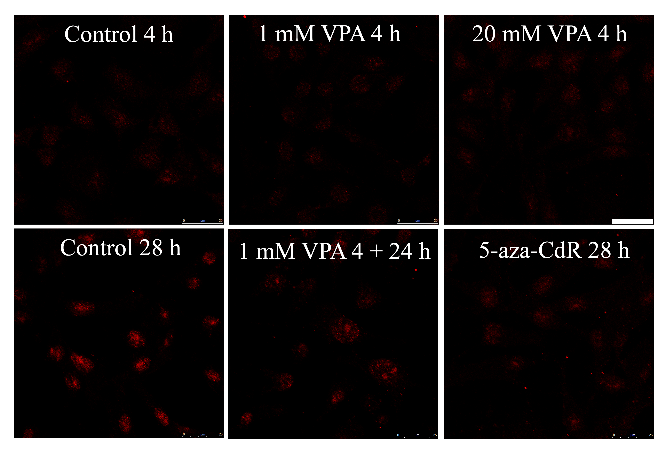

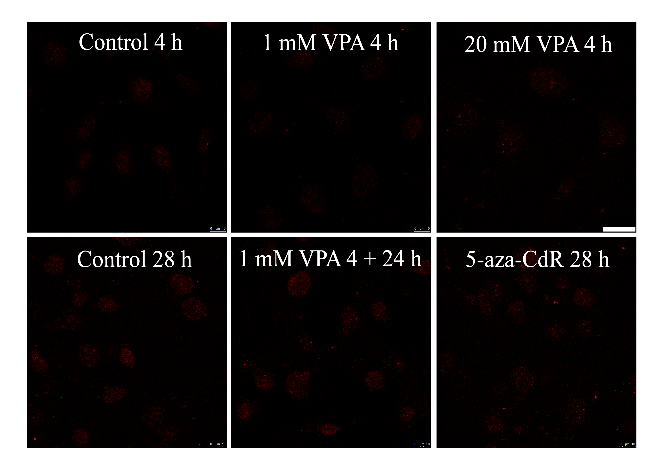


**a**

**b**

**Supplementary Figure S1. 5caC and 5fC abundance in VPA- and 5-aza-CdR-treated HeLa cells arrested in the G1 phase.** Unchanged abundance of 5caC and 5fC is evident in treated cells compared to respective controls. Scale bars indicate 20 μm (a) and 25 μm (b). Images are representative of two experiments.


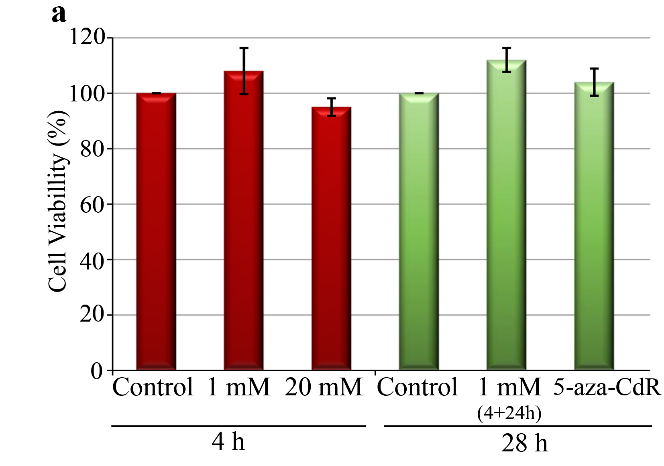

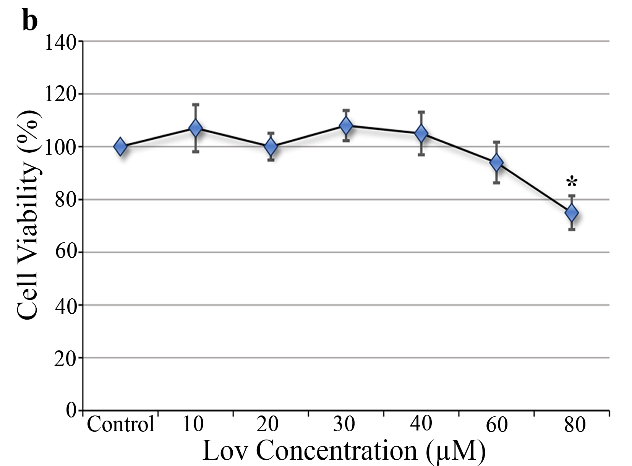


**Supplementary Figure S2. Determination of cell viability of HeLa cells using the MTT assay.** No change in cell viability resulted in response to VPA- and 5-aza-CdR- treatments (a). The cell viability was significantly reduced after treatment with 80 µM lovastatin (Lov) (b). Data represent means and standard deviations of four (a) and three (b) independent experiments. Using Student’s t-test, a significant difference at P <0.05 was demonstrated (*).


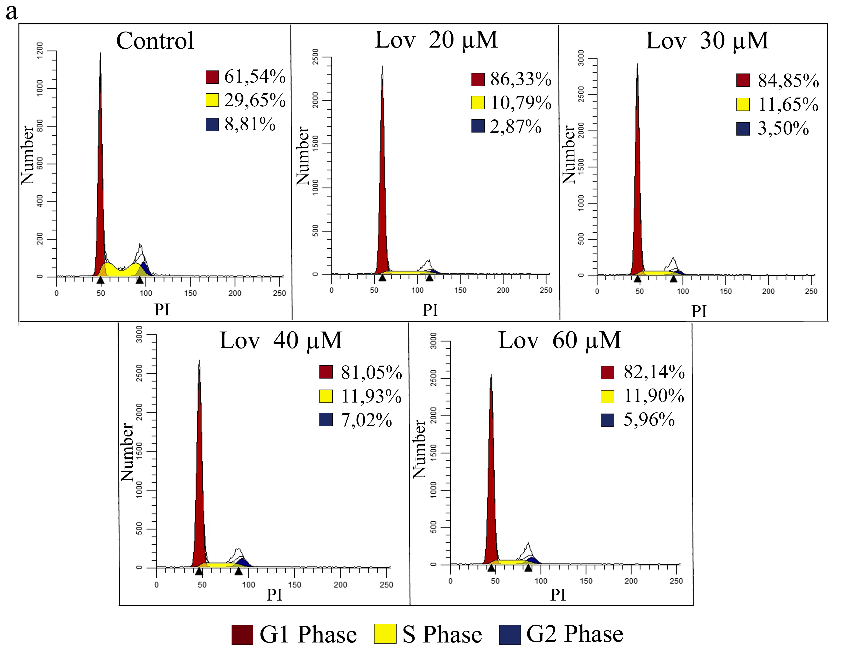


**a**


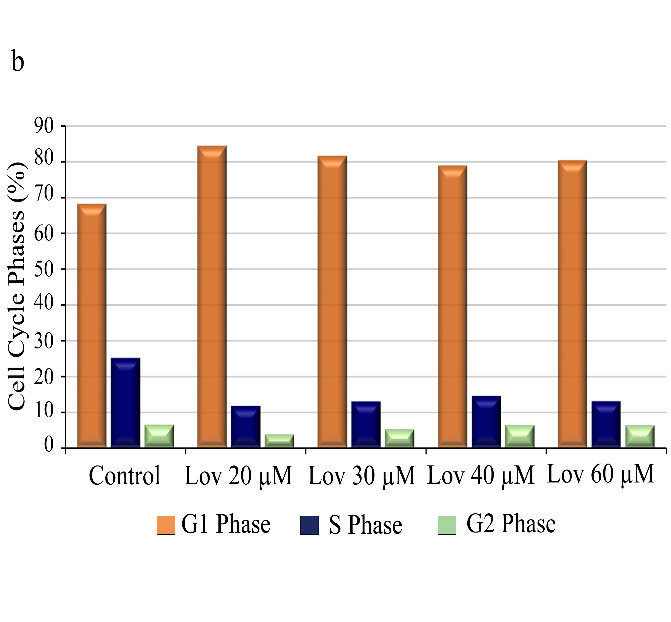


**b**

**c**


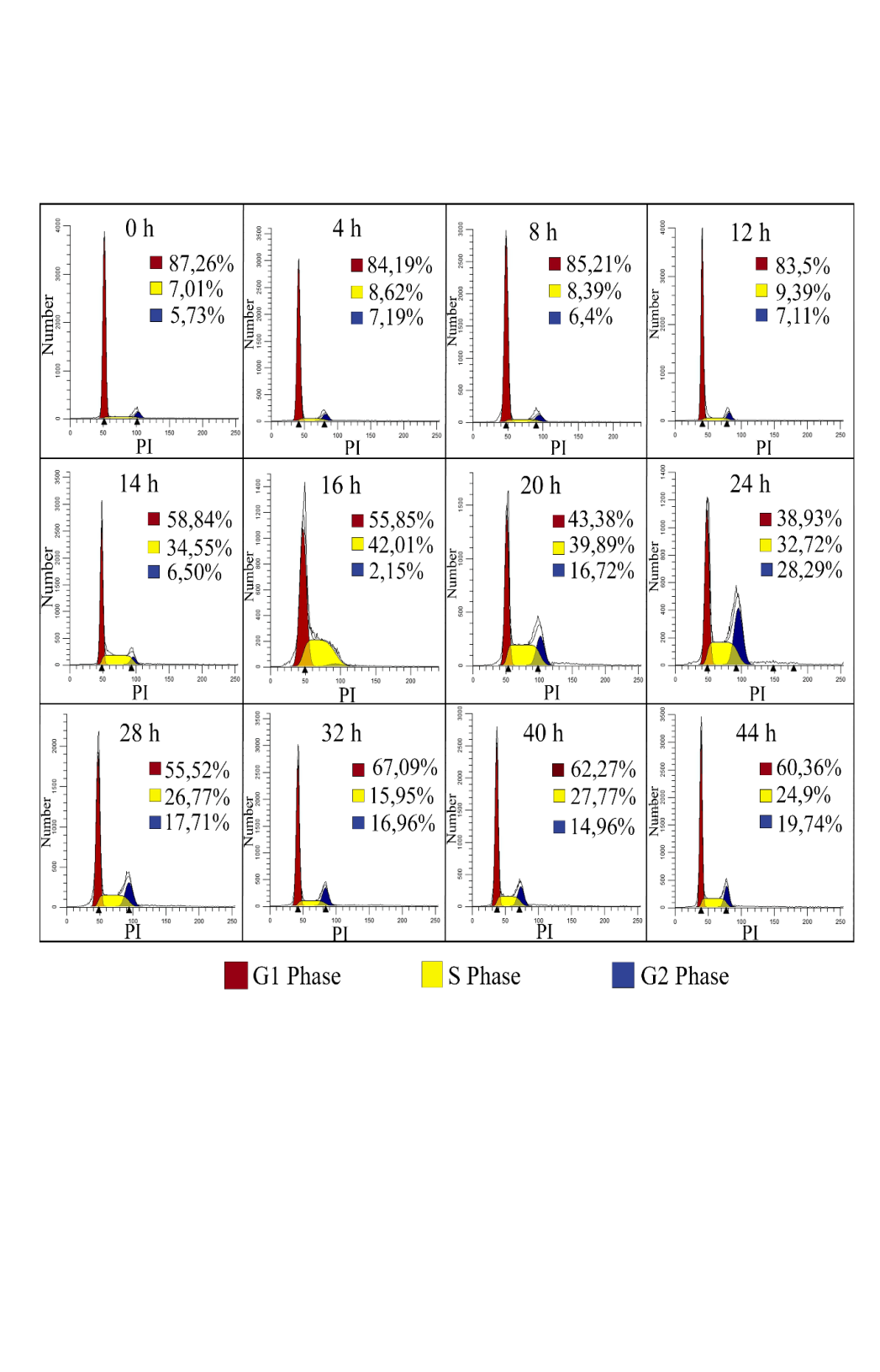


**Supplementary Figure S3. Effects of lovastatin (Lov) and mevalonic acid treatments on HeLa cells.** The optimal Lov concentration sufficient to obtain a higher frequency of cells in G1 phase (20 µM) is shown (a, b). The optimal time duration of 16 h was sufficient to obtain the highest number of cells synchronized in the S phase (c). Mean and standard error data are representative of three (a, b) and two (c) independent experiments.

**
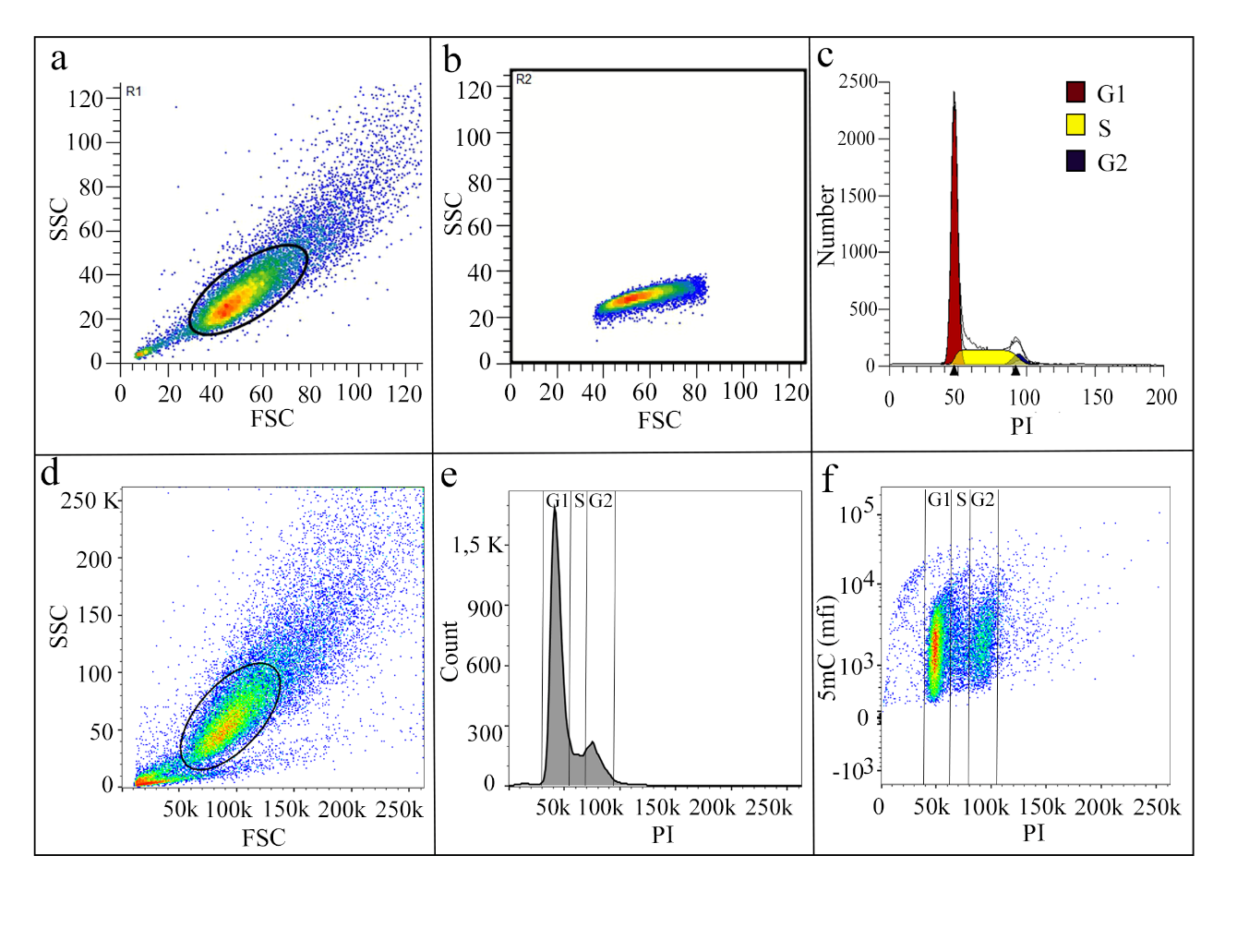
**

**Supplementary Figure S4. Distribution of cell cycle phases and quantification of 5mC in HeLa cells using ModFit LT ™ and FlowJo® softwares.** PI-stained cells were selected according to their forward scatter (FSC) and side scatter (SSC) (a, b). Cell cycle phases were determined using PI scale (c). To quantify 5mC content, HeLa cells were labelled with anti-5mC antibody prior to DNA PI staining and selected according to their FSC x SSC values (d). PI scale was used to discriminate the cell cycle phases (e) which were associated with 5mC (f) content. 5mC mean fluorescence intensity (mfi) was obtained at each cell cycle phase.


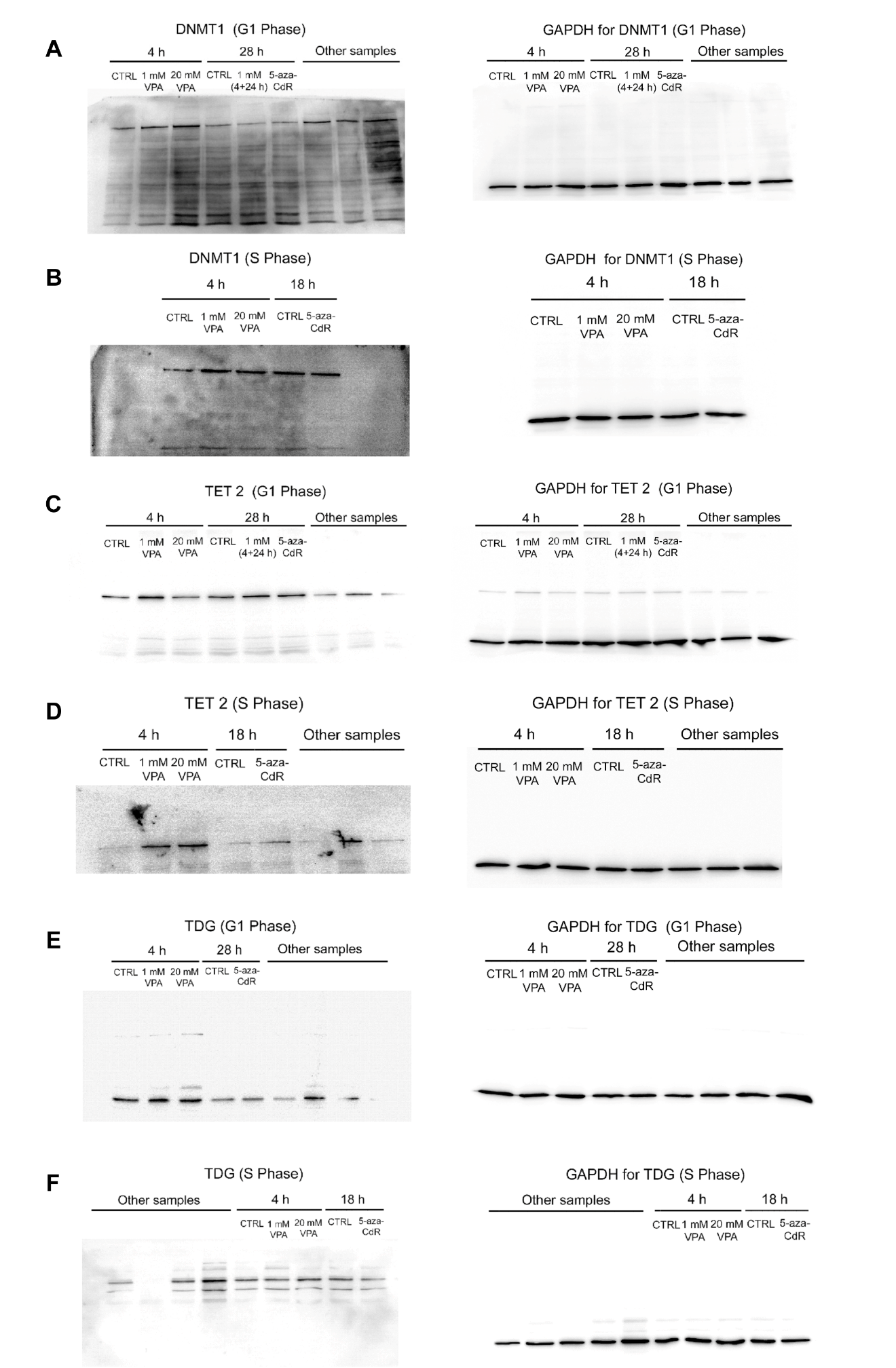
**Supplementary Figure S5.** Full-length blots that refer to Figure 4 of the manuscript. A, B, C, D, E and F, correspond to images a, b, c, d, e and f, respectively, of Figure 4.
